# Study of Rotation and Bending Effects on a Flexible Hybrid Implanted Power Transfer and Wireless Antenna System

**DOI:** 10.3390/s20051368

**Published:** 2020-03-02

**Authors:** Reem Shadid, Mohammad Haerinia, Sima Noghanian

**Affiliations:** 1 Electrical Engineering Department, Applied Science Private University, Amman 11931, Jordan; re_shadid@asu.edu.jo; 2 School of Electrical Engineering and Computer Science, University of North Dakota, Grand Forks, ND 58202, USA; mohammad.haerinia@und.edu; 3 Phoenix Analysis and Design Technologies, Tempe, AZ 85284, USA

**Keywords:** wireless power transfer, bending effects, misalignment, hybrid power transfer

## Abstract

We present rotational misalignment and bending effects on a hybrid system to transfer power and data wirelessly for an implantable device. The proposed system consists of a high-frequency coil (13.56 MHz) to transfer power and an ultra-high frequency antenna (905 MHz) for data communication. The system performance and the transmitted power were studied under two misalignment conditions: (1) receiver rotation around itself with reference to the transmitter, and (2) bending of the implanted receiver under three different radii. Implanted receiver was printed on a flexible Kapton substrate and placed inside a layered body tissue model at a 30 mm depth. It is shown that the inductive link is stable under rotational misalignment and three bending conditions, whereas the communication data link is suitable to be used if the rotation angle is less than 75° or larger than 150°. The results show that the resonance frequency varies by 1.6%, 11.05%, and 6.62% for the bending radii of 120 mm, 80 mm, and 40 mm, respectively. Moreover, transmission efficiency varies by 4.3% for the bending radius of 120 mm. Decreasing the bending radius has more effects on antenna transmission efficiency that may cause severe losses in the communication link.

## 1. Introduction

Recently, industrial and academic researchers have shown significant interest in the field of designing unified systems consisting of wireless power transfer (WPT) and a communication data links, especially for biomedical applications. 

In the last few years, research on implanted and flexible electronics has been focused on electrodes, substrate materials, and relevant electronic components. The authors of Reference [1] presented a slot-patch flexible antenna working at 434 MHz, used as an implant for animals and ingestible for humans. In Reference [2], the authors present a near-field capacitive coupling to transfer 100 mW wirelessly to a flexible power receiver. However, using a capacitive coupled powering scheme needs to be investigated for long term subcutaneous implant applications to limit its harmful effect. In Reference [3], the authors designed a fully implantable stimulator with WPT and data transmission. Their design consisted of Litz wire and it was not flexible. The authors in Reference [4] fabricated a flexible printed dual-band dipole antenna, operating at 900 MHz/2.44 GHz, a low-cost fabrication process. This antenna was considered a receiving antenna and was connected to a rectifier. The authors in References [5,6] analyzed the efficiency and investigated the effects of the shape on magnetic flux density in wireless power transfer systems. The authors in Reference [7] designed a printed dipole antenna on a flexible substrate for the ultra-high frequency (UHF) band. The total dimension was 120 mm × 50 mm × 0.05 mm. 

The critical challenge in implantable devices is miniaturization. To minimize the footprint of the implanted wireless system, we introduced a hybrid system that combines coils used for wireless power transfer at 13.56 MHz and antennas for data communication at 905 MHz. The hybrid system was presented in Reference [8]. The implanted system was named “IM”, and the external antenna/coil combination, which was placed on the skin layer outside the body, was named “EX”. Ideally, the maximum coupling occurred when EX and IM were perfectly aligned. However, various misalignments may occur. From Reference [9], we studied the effect of lateral and angular misalignments on the hybrid system design. We analyzed the misalignment effects on the link budget in Reference [10]. 

The rotational misalignment and bending are two conditions that may happen because of changes in the IM location due to body movement or inaccuracy in the placement of the EX. Moreover, bending of the IM can easily occur due to the person’s movement or anatomical conditions, such as skin mobility and variations in the thickness of subcutaneous fatty tissue [11]. Additionally, conformal and flexible shapes are of interest for implanted devices in order to minimize the discomfort and scar effects, which may require bending of the IM. This work focuses on the study of the rotational misalignment and bending effect on the IM performance. 

The paper is organized as follows. Section 2 provides the antenna and coil design. The rotational misalignment study based on numerical simulation is presented in Section 3. Bending effects were studied numerically in Section 4. In Section 5, fabrication and measurements results are provided. Conclusions are summarized in Section 6. Information about phantom fabrication and measurement is given in Appendix A.

## 2. Design

**Figure 1:**
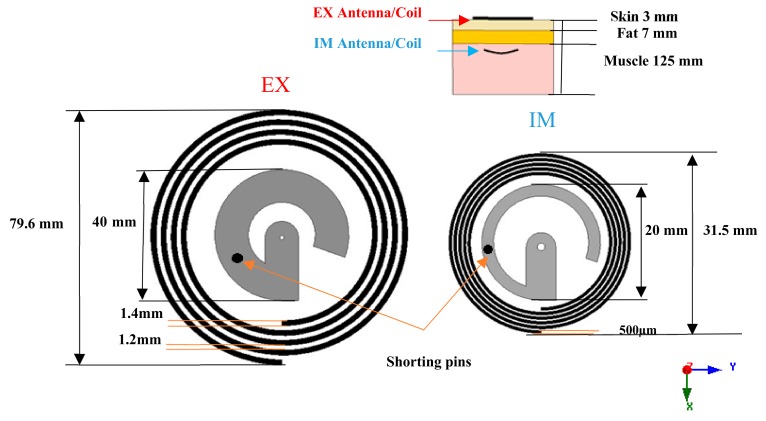
Coil and antenna configuration for the external antenna/coil combination (EX) and implanted system (IM) and their location, with respect to the layered body model.

The proposed system consisted of a hybrid coil/antenna named “EX”, which was placed directly at the outer surface of the body, and another coil/antenna system named “IM”, which was embedded inside a layer of muscle at 30 mm distance away from the EX. The EX and IM microstrip antennas had a G-shape profile (Figure 1). 

The coils had a spiral shape. Details of the design and optimization of the dimensions of the EX and IM are presented in Reference [12]. IM was considered to be printed on flexible Kapton material. Kapton was chosen due to its specification, and, in addition to its flexibility and biocompatibility, it has a low loss factor over a wide frequency range, high tensile strength, and dielectric strength [13]. This Kapton substrate (ε_r_ = 3.9, σ = 0.00524 siemens/m) is considered to be covered by a layer of silicone material. Silicon is biocompatible and is needed for an implanted device. A drawing of the hybrid system design is shown in Figure 1. The location of the shorting pin was chosen such that it tuned the EX and IM antennas to operate at 905 MHz. The locations of the shorting pins were optimized for achieving the maximum power efficiency for the selected antenna dimension parameters for both EX and IM parts, and the results are summarized in Table 1. The origin of the coordinate system was assumed to be at the antenna’s center. 

The body was modeled as a layered structure of skin, fat, and muscle. The electromagnetic properties of each layer at each frequency were obtained from the Institute of Applied Physics (IFAC) database and are summarized in Table 2. The thickness of the layers is shown in Figure 1. 

## 3. Rotational Misalignment

**Figure 2:**
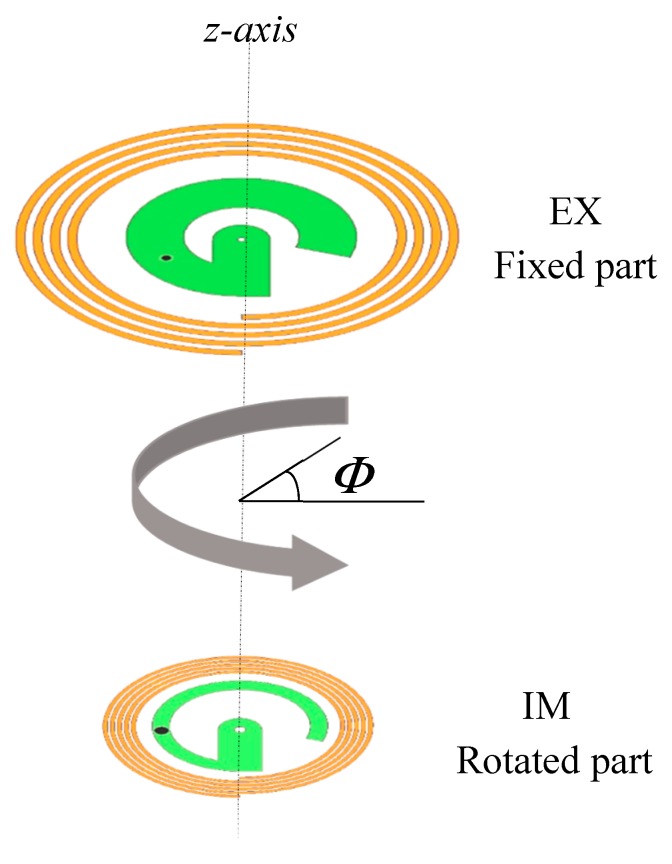
Rotational misalignment setting.

The WPT technique, based on the inductive links, is one of the promising solutions for powering biomedical implanted devices. Ensuring a stable power transfer and data communication in implanted device, under conditions, such as misalignment, is challenging. Therefore, the performance of our proposed hybrid system is investigated and studied in two cases: 

First, since the G-shape was not symmetrical in the xy plane, we needed to investigate the performance of the hybrid system by rotating the IM combination around the z-axis while keeping the EX plane unchanged, as shown in Figure 2. The rotating angle (*Φ*) was changed from 0° to 360°. The distance between the EX and IM combinations was kept at 30 mm. This study was based on simulation. For this part of the study, the Kapton layer was considered to have 0.8 mm thickness.

We studied the changes in the coupling coefficient k, reflection coefficients S_11_ (Tx), S_22_ (IM), transmission coefficient S_21_, and transmission efficiency *η*. ANSYS HFSS (high frequency structure simulator) software was used to simulate the G-shape antenna, whereas ANSYS Maxwell was used for simulating the coils around 13.56 MHz, then the simulated design was imported to ANSYS Twin Builder to calculate S_21_ and *η* = |S_21_|^2^ × 100%. All simulations were done using the 2019R2 version of the above-mentioned simulation tools. 

**Figure 3:**
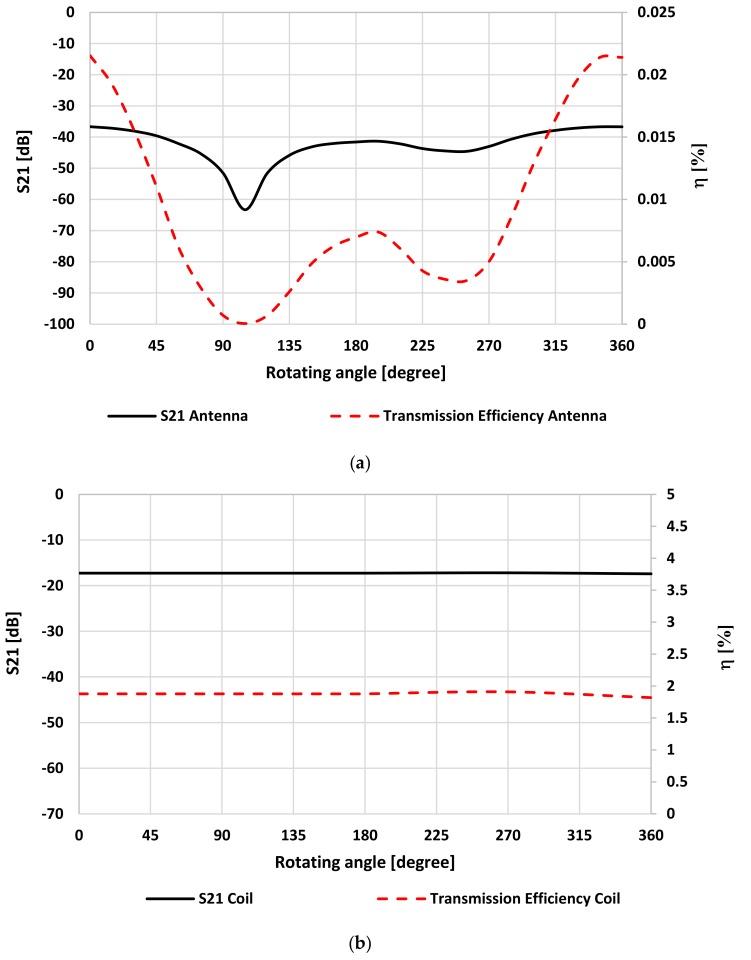
S_21_ and *η* under rotational misalignment for the IM around z axis (the IM was at 30 mm depth) for (**a**) the antenna pair and (**b**) the coil pair.

The results of the simulation of the bending scenario are shown in Figure 3. These graphs show the relationship between S_21_ and *η* versus *Φ* for antenna and coil pairs when the IM was rotated around the z-axis. The tissue model shown in Figure 1 was used. It is noted that S_21_ and *η* for the coil set were not affected by angular misalignment due to their symmetrical shape. However, S_21_ values for the antenna pair were more stable (S_21_ varied around 20% from the perfectly aligned case) for misalignment angles less than 75° or more than 150°. If the angles were not within these ranges of S_21_ drops, the worst case occurred at 105°.

## 4. Bending 

**Figure 4:**
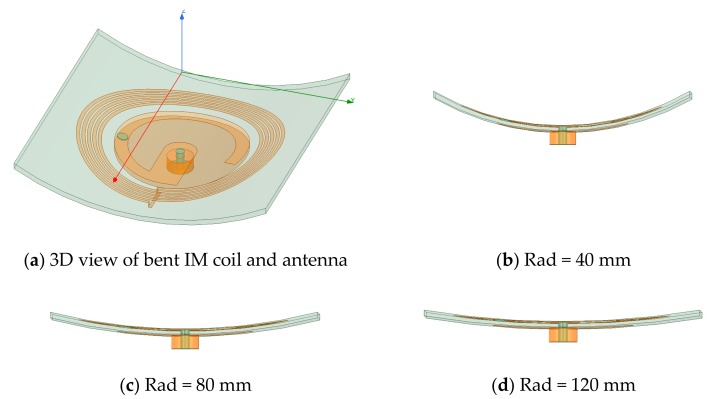
**The** IM hybrid design under three bending conditions. (**a**) three dimensional views of bent RX, (**b**) Rad = 40 mm, (**c**) Rad = 80 mm, and (**d**) Rad = 120 mm.

**Figure 5:**
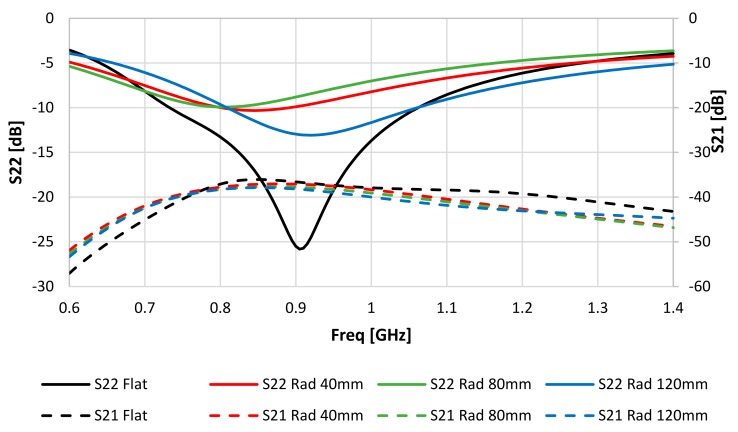
Simulated S_21_ and S_22_ for the IM under three bending cases (Kapton thickness of 0.8 mm).

The impact of bending the IM coil/antenna on the performance was investigated under three bending conditions (Rad = 40 mm, 80 mm, and 120 mm), as shown in Figure 4. Figure 5 shows the simulation results of the effects of bending on the EX and IM performance. 

Figure 5 shows the simulation results of S_22_ and S_21_ for the IM antenna based on the tissue model shown in Figure 1 under bending conditions. Table 3 shows these parameters at the resonance frequency for various bending radii. It can be noted that the IM resonance frequency (f_oIM_) varied as much as –100 MHz at Rad = 80 mm to +15 MHz at Rad = 120 mm, compared to its value at f_oIM_ for the flat case. Please note that the larger Rad value, the closer the coil was to the flat case. 

While S_21_ did not vary more than 1.57 dB, bending the IM had significant effects on the resonance frequency of the antenna and the level of S_22_. This value was increased by 15.51 dB, 15.89 dB, and 12.74 dB at Rad values of 40 mm, 80 mm, and 120 mm, respectively. Moreover, the –9 dB bandwidths of antennas are given in Table 3. It can be seen the maximum bandwidth happened at Rad = 120 mm.

Table 4 shows coupling (k) values under all bending condition cases at 13.56 MHz. It can be noted that bending had a very small and negligible effect on the performance of the inductive link. 

## 5. Fabrication and Measurements

**Figure 6:**
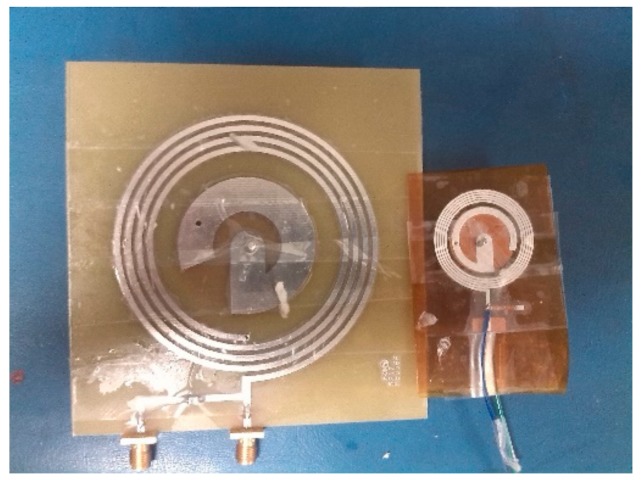
The fabricated prototype of the EX (left) and IM (right).

The EX and IM were fabricated using a Voltera V-One automated printed circuit board (PCB) printer system [14]. A flexible conductive ink was used that was suitable for printing on a flexible substrate, such as Kapton (DuPont, Wilmington, DE, USA) (polyimide), polycarbonate, and polyethylene terephthalate (PET). This specific ink had a curing temperature of 140 °C for 10 min or 120 °C for 30 min of curing. The printed prototypes of the EX and IM are shown in Figure 6. To create it, the shorting pin a hole was created by drilling and filled by the same conductor ink. It is important to note that we were not able to obtain Kapton at 0.8 mm. The thickness of the Kapton substrate used for measurement was 0.15 mm. To compare the results, a new set of simulations was conducted based on the corresponding design. While the thickness had no effect on the coil operation, it may have caused changes in the frequency of the antenna. 

To mimic the effect of tissue on the implanted system, we used a muscle phantom. A phantom is a tissue mimicking material. In general, phantoms should represent the dielectric permittivity and conductivity (losses) of the tissue of interest at the working frequency. The muscle phantom was created based on ingredients and steps described in Reference [15] to represent the muscle tissue model; more details are mentioned in Index I. 

**Figure 7:**
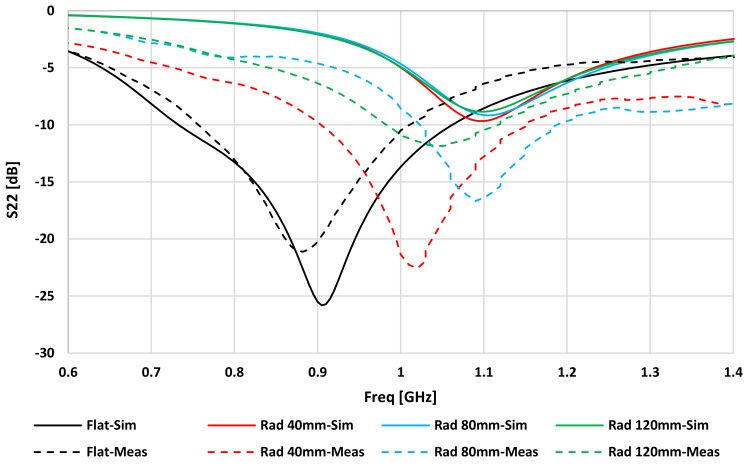
Simulated and measured IM reflection coefficients (S_22_) for flat and three bending cases (Kapton thickness of 0.15 mm).

**Figure 8:**
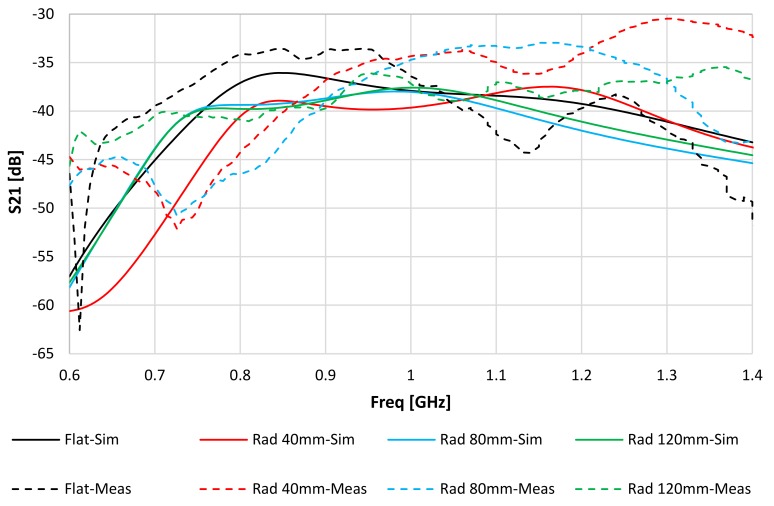
Simulated and measured transmission coefficient (S_21_) for flat and three bending cases (Kapton thickness of 0.15 mm).

Figure 7 and Figure 8 show the simulated and measured values of S_22_ and S_21_ for the IM antenna, respectively. There is a small difference between the measured and simulated resonance frequencies. S_22_ varied from 1.35% at Rad = 80 mm to 6.84% at Rad = 40 mm. This could be because the IM was not completely covered by the phantom in the back of the hybrid design. 

## 6. Conclusions

In this paper, we studied the rotation misalignment and bending effects on the performance of a hybrid power transfer and communication system design. Two main conclusions are as follows. First, for the inductive link, the system was not affected by angular misalignment because of the symmetry of the coils. Additionally, the bending did not have much of an effect on the coil system. Second, for the data communication link through antennas, the system performance was acceptable if the rotation angle was less than 75° or larger than 150°. Moreover, the results show that the resonance frequency varied from the flat shape by 1.6%, 11.05%, and 6.62% at Rad = 120 mm, 80 mm, and 40 mm, respectively, whereas the maximum variation of S_21_ was around 4.3% at Rad = 120 mm. However, increasing the bending (decreasing Rad) may have caused severe losses in the communication link and is not recommended.

## Figures and Tables

**Table 1:** Shorting pin location for 905 MHz resonance.

Antenna Location	x (mm)	y (mm)
EX	6.75	−13
IM	0.30	−9

**Table 2:** Layered body tissue assumed electromagnetic properties at different frequencies.

Frequency	13.56 MHz			905 MHz		
Tissue	Muscle	Fat	Skin	Muscle	Fat	Skin
ε_r_	138.4	11.83	285.25	55	5.46	41.4
Loss tangent	6.01	3.40	1.11	0.34	0.19	0.42

**Table 3:** Antenna characteristics under bending conditions.

Rad (mm)	f_0IM_(MHz)	S_22_(dB)	S_21_(dB)	Bandwidth (Hz)
40	845	−10.31	−37.12	202
80	805	−9.93	−37.80	148
120	920	−13.08	−38.25	318
flat	905	−25.82	−36.68	372

**Table 4:** Coils coupling coefficient (k) under bending conditions.

Rad (mm)	k
40	0.054
80	0.052
120	0.054
flat	0.057

**Figure A1:**
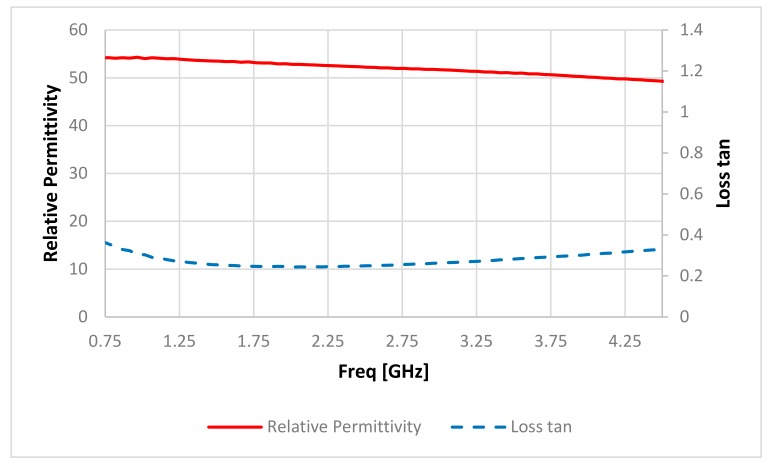
Measured relative permittivity and loss tangent of fabricated muscle phantom.

## Appendix A. Phantom Ingredients and Fabrication (Muscle Type)

*a. Muscle Phantom Ingredients and Permittivity Measurements:*

Table A1 summarizes the material type and amount needed for muscle phantom fabrication. The phantom material was measured at room temperature using a Keysight 85070E dielectric probe. The results are shown in Figure A1.

**Table A1:** Ingredients of muscle tissue-mimicking phantom.

Type	Water	Gelatin	NaCl	Oil	Detergent Ultra Ivory	Food Coloring
**Ingredients (g)**	230	34.1	1.2	35	40	1.3

*b. Fabrication process:*

The fabrication of the muscle phantoms requires the following steps:

1. Mix the gelatin and 100 g de-ionized water in a beaker.
2. Cover the beaker with cling film and heat the mixture up to 80 °C in a hot water jacket or in a double boiler.
3. Leave the mixture to cool until 35 °C.
4. Add the remaining room temperature deionized water and NaCl whilst stirring the mixture slowly.
5. Add the room temperature dish-washing detergent to the mixture and keep stirring slowly.
6. When the mixture reaches 28 °C, add room temperature oil and keep stirring slowly until the mixture becomes homogeneous. At this point, the color of the mixture changes to white. We can also add food coloring at this point.
7. Pour the homogeneous mixture into containers and leave it to solidify overnight.

Stir the mixture slowly to avoid air bubbles when making the tissue-mimicking phantoms. Moreover, the mixture should be handled with care.
